# Surfactin facilitates establishment of *Bacillus subtilis* in synthetic communities

**DOI:** 10.1093/ismejo/wraf013

**Published:** 2025-01-23

**Authors:** Carlos N Lozano-Andrade, Caja Dinesen, Mario Wibowo, Nil Arenos Bach, Viktor Hesselberg-Thomsen, Scott A Jarmusch, Mikael Lenz Strube, Ákos T Kovács

**Affiliations:** DTU Bioengineering, Technical University of Denmark, 2800 Kgs Lyngby, Denmark; DTU Bioengineering, Technical University of Denmark, 2800 Kgs Lyngby, Denmark; Institute of Biology, Leiden University, 2333 BE Leiden, The Netherlands; DTU Bioengineering, Technical University of Denmark, 2800 Kgs Lyngby, Denmark; DTU Bioengineering, Technical University of Denmark, 2800 Kgs Lyngby, Denmark; DTU Bioengineering, Technical University of Denmark, 2800 Kgs Lyngby, Denmark; DTU Bioengineering, Technical University of Denmark, 2800 Kgs Lyngby, Denmark; DTU Bioengineering, Technical University of Denmark, 2800 Kgs Lyngby, Denmark; DTU Bioengineering, Technical University of Denmark, 2800 Kgs Lyngby, Denmark; Institute of Biology, Leiden University, 2333 BE Leiden, The Netherlands

**Keywords:** secondary metabolites, synthetic community, *Bacillus subtilis*surfactin, establishment, chemical ecology

## Abstract

Soil bacteria are prolific producers of a myriad of biologically active secondary metabolites. These natural products play key roles in modern society, finding use as anti-cancer agents, as food additives, and as alternatives to chemical pesticides. As for their original role in interbacterial communication, secondary metabolites have been extensively studied under *in vitro* conditions, revealing many roles including antagonism, effects on motility, niche colonization, signaling, and cellular differentiation. Despite the growing body of knowledge on their mode of action, biosynthesis, and regulation, we still do not fully understand the role of secondary metabolites on the ecology of the producers and resident communities *in situ.* Here, we specifically examine the influence of *Bacillus subtilis*-produced cyclic lipopeptides during the assembly of a bacterial synthetic community, and simultaneously, explore the impact of cyclic lipopeptides on *B. subtilis* establishment success in a synthetic community propagated in an artificial soil microcosm. We found that surfactin production facilitates *B. subtilis* establishment success within multiple synthetic communities. Although neither a wild type nor a cyclic lipopeptide non-producer mutant had a major impact on the synthetic community composition over time, both the *B. subtilis* and the synthetic community metabolomes were altered during co-cultivation. Overall, our work demonstrates the importance of surfactin production in microbial communities, suggesting a broad spectrum of action of this natural product.

## Graphical Abstract

**Graphical Abstract:**
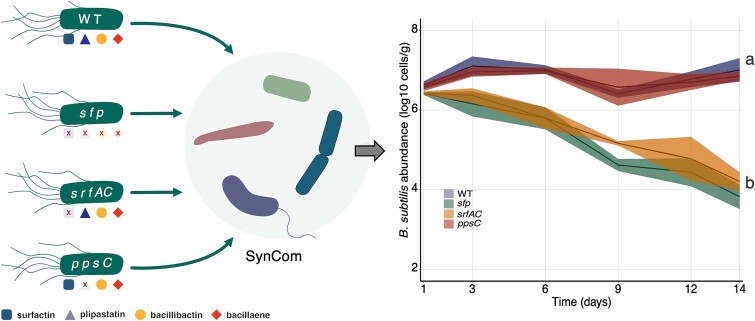

## Introduction

Microbes produce a plethora of small molecules with diverse activities, which are extensively exploited in modern society. Several of these natural products, often denoted as secondary or specialized metabolites (SMs), have been pivotal in contemporary medicine and biotechnological industries [1, 2]. They serve as frontline therapy against infectious diseases, therapeutics for cancer [1], food additives [3], or crop protection agents [4, 5]. Besides the long-standing tradition of industrial exploitation, SMs are considered chemical mediators that modulate interactions within and between microbial species or even cross-kingdoms. For instance, defensive molecules might help producers defend their resources or niche from microbial competitors [6]. Furthermore, some SMs function as signal molecules for coordinated growth (i.e. for quorum-sensing) [7, 8] and cell-differentiation [9, 10].

Among the diverse array of SM-producing microorganisms, the *Bacillus subtilis* species complex stands out as a prolific group with significant potential for SM production. This soil-dwelling bacterial species comprises several strains capable of synthesizing a wide range of SMs, including cyclic lipopeptides (LPs), polyketides, ribosomally synthesized and post-transcriptionally modified peptides, and signaling molecules [11–15]. Specifically, LPs are the most extensively studied class. They are synthesized by non-ribosomal peptide synthase (NRPS), acting as a molecular assembly line that catalyzes the incorporation of amino acids into a growing peptide [16]. In the *B. subtilis* species group, LPs are structurally categorized into three families: surfactins, iturins, and fengycins, based on their peptide core sequence. These molecules consist of seven (surfactins and iturins) or ten (fengycins) α-amino acids linked to β-amino (iturins) or β-hydroxyl (surfactin and fengycins) fatty acids [17, 18]. LPs exemplify multifunctional SMs, acting not only as antimicrobials by antagonizing other microorganisms but also playing pivotal roles in processes including motility, cellular differentiation, surface colonization, and signaling [11, 19–25].

Although significant progress has been made in understanding the mode of action, biosynthesis, regulation, and functionality of LPs, their natural functions in natural environments remain largely uncharacterized. Experimental studies addressing these questions are constrained by the immense biological and chemical diversity of soil microbiomes and the community-level interactions modulating SMs functions. Additionally, technical challenges in tracking and quantifying the *in situ* productions of LPs and other classes of SM pose further barriers to elucidating their natural role in soil [14, 26–29].

Most evidence supporting the multifaceted functions of LPs has been gathered under *in vitro* conditions using pure cultures. However, these controlled settings may not accurately reflect the complexity of soil environments and the actual dynamics of SMs production in a broader ecological context. To address this limitation, several studies have adopted the use of less complex systems that mimic natural biomes [30, 31]. One promising strategy is the use of synthetic bacterial communities (SynComs), which allow for the testing of fundamental ecological questions in controlled yet more ecologically relevant conditions [32, 33]. For instance, Cairns *et al.* used a 62-strain SynCom to demonstrate how low antibiotic concentration impacts community composition and horizontal transfer of resistance genes, whereas Niu *et al.* built a seven-member community mimicking the core microbiome of maize, which was able to protect the host from a plant-pathogenic fungus [34, 35]. Simultaneously, the development of soil-like matrices and artificial soil has provided a useful option for studying chemical ecology in highly controlled gnotobiotic systems compatible with analytical chemistry and microbiological methods [36–38]. Thus, coupling the use of artificial soil systems and simplified SynCom is a fast-growing approach to examine microbial interactions whereas maintaining some degree of ecological complexity.

This study aims to explore the roles of LPs produced by a *B. subtilis* isolate [11, 39, 40] during SynCom assembly and simultaneously dissect the impact of LPs on *B. subtilis* establishment success within SynComs. Utilizing an artificial soil-mimicking system [41, 42], we assessed the impact of non-ribosomal peptides and bacillaene (a hybrid NRPS – polyketide) (s*fp*), as well as specifically surfactin (*srfAC*) or plipastatin (*ppsC*), on the ability of *B. subtilis* to establish within a four-member SynCom. We demonstrated that surfactin production facilitates *B. subtilis* establishment success within SynCom in a soil-mimicking environment. Regarding the SynCom assembly, we found that the wild-type and non-producer strains had a comparable influence on the SynCom composition over time. Moreover, we revealed that the *B. subtilis* and SynCom metabolome were both altered. Intriguingly, the importance of surfactin for the establishment of *B. subtilis* has been demonstrated in diverse SynCom systems with variable composition. Altogether, our work expands the knowledge about the role of surfactin production in microbial communities, suggesting a broad spectrum of action of this natural product.

## Materials and methods

### Bacterial strains and culture media

All the strains used in this study are listed in Table S1. *B. subtilis* strains were routinely grown in lysogeny broth (LB) medium supplemented with the appropriated antibiotic (LB-Lennox, Carl Roth, Karlsruhe, Germany; 10 g/L tryptone, 5 g/L yeast extract, and 5 g/L NaCl) at 37°C with shaking at 220 rpm. The strains composing the different synthetic communities were grown in 0.5 × Trypticase Soy Broth (TSB; Sigma-Aldrich, St. Louis, Missouri, USA) for 24 h at 28°C with shaking at 220 rpm.

### *Bacillus subtilis* establishment in the Dyrehaven synthetic community propagated in soil-like matrix

The impact of introducing *B. subtilis* P5_B1 and its secondary-metabolite-deficient mutants into the SynCom was investigated using an artificial soil-mimicking microcosm [42]. Spherical beads were created by dripping a polymer solution, comprising 9.6 g/L of Phytagel™ and 2.4 g/L sodium alginate in distilled water, into a 2% CaCl2 cross-linker solution [41]. After 2 h of soaking in 0.1× TSB as a nutrient solution, the beads were sieved to remove any residual medium. Twenty milliliters of beads were then transferred to 50 ml Falcon tubes. Cultures of *B. subtilis* P5_B1 and the four SynCom members were grown as described above. The members of the SynCom were mixed at different OD, whereas fast-growing strains (i.e. *S. indicatrix* and *Chryseobacterium* sp.) had to be included at low density to ensure SynCom stability. Specifically, *Pedobacter* sp. and *Rhodococcus globerulus* were adjusted to OD 2.0, whereas *S. indicatrix* and *Chryseobacterium* sp. were adjusted to OD 0.1 before mixing. Suspensions of *B. subtilis* P5_B1 and its mutants were standardized to OD 2.0. Next, bacterial inocula were prepared by mixing equal volumes of these adjusted cultures (four members plus each *B. subtilis* strain, respectively), and 2 ml of this suspension was then inoculated into freshly prepared beads. The bead microcosms were statically incubated at room temperature. Concurrently, microcosms inoculated with each strain as a monoculture were set as controls. At days 1, 3, 6, 9, 12, and 14, one gram of beads was transferred into a 15 ml Falcon tube, diluted in 0.9% NaCl, and vortexed for 10 min at maximum speed to disrupt the beads. The suspensions were then used for cell number estimation via colony-forming unit (CFU) and flow cytometry. For colony counting, 100 μL of the sample was serially diluted, spread onto 0.1× TSA, and CFU were estimated after 3 days. For the quantification of *B. subtilis* using flow cytometry, the samples were first passed through a Miracloth (Millipore) to remove any trace of beads and diluted 100-fold in 0.9% NaCl. Subsequently, 1 ml of each sample was transferred to an Eppendorf tube and assayed on a flow cytometer (MACSQuant VYB, Miltenyi Biotec). *gfp*-labeled *B. subtilis* was detected using the blue laser (488 nm) and filter B1 (525/50 nm). Cells above 1 cell/ml were detected. Controls with non-inoculated beads and 0.1× TSB were employed to identify background autofluorescence. Single events were gated into the GFP vs. SSC-A plot, where GFP-positive cells were identified for each sample.

### WT:*srfAC* complementation assay

Overnight cultures of the strains of interest (OD600 = 2.0; WT::mKate and *srfAC*::gfp) were premixed at 1:1 ratio. The inoculum was prepared by mixing equal volumes of the premixed *Bacillus* suspension with each member of the SynCom. Subsequently, 2 ml of this mixture were inoculated into freshly prepared beads. Propagation of the microcosms and *B. subtilis* quantification were performed as described above.

### Detection of secondary metabolites from artificial soil microcosms

To extract secondary metabolites from the bead samples, 1 g of bead was transferred into a 15 ml with 4 ml of isopropyl alcohol:ethyl acetate (1:3 v/v), containing 1% formic acid. The tubes were sonicated for 60 min and centrifuged at 13400 rpm for 3 min. Then, the extracts were evaporated under N_2_ overnight, re-suspended in 300 μL of methanol, and centrifuged at 13400 rpm. The supernatants were transferred to an HPLC vial and subjected to ultrahigh-performance liquid chromatography-high resolution mass spectrometry (UHPLC-HRMS) analysis. The running conditions and the subsequent data analysis were performed as previously described [42].

### Metatranscriptomic analysis

For the RNA sequencing, the SynCom was propagated in the artificial soil matrix and challenged with either *B. subtilis* P5_B1 or the mutant impaired in NRP synthesis (*sfp* mutant). A SynCom without *B. subtilis* inoculation served as the control group. On days 1 and 6, 4 g of beads from each treatment were snap-frozen in liquid nitrogen and stored at −80°C. The RNA extraction was performed using the RNeasy PowerSoil Total RNA Kit (QIAGEN) following the manufacturer’s instructions. After extraction, the samples were treated with the TURBODNA-free kit (ThermoFisher) to degrade the remaining DNA. The library preparation and sequencing were carried out by Novogene Europe on a NovaSeq 6000 S4 flow cell with PE150 (Illumina).

The reads were demultiplexed by the sequencing facility. Subsequently, reads were trimmed using Trimmomatic v.0.39 [43]. Quality assessment was performed using FASTQC, and reads were sorted with SortMeRNA v.4.2.0 [44] to select only the non-rRNA reads for the downstream analysis. Reads were then mapped onto the genomes of the strains (D764, D763, D757, D749, and *B. subtilis* P5_B1) using Bowtie v.2–2.3.2 [45]. Differential gene expression analysis was conducted using the R package DESeq2 [46] using the shrunken log2 fold change values for analysis [47] The *P* values of each gene were corrected using Benjamini and Hochberg’s approach for controlling the false discovery rate (FDR). A gene was considered as differentially expressed when absolute log2 fold change was greater than 2 and FDR was less than 0.05. For functional analysis, the protein-coding sequences were mapped with KEGG Ontology, Gene Ontology (GO) terms, and Clusters of Orthologous Genes (COGs) using eggNOG-mapper [48]. Then, the eggNOG-mapper annotated dataset was used for gene set enrichment for pathway analysis in GAGE [49]. Transcriptomic analysis was performed from three independent replicates for each sample.

### Inhibition assay

The *in vitro* antagonistic effect of *B. subtilis* P5_B1 and its secondary metabolite-deficient mutants was assessed using double-layer agar plate inhibition assays against each SynCom member (target bacterium). All strains were cultured for 24 h in 0.1× TSB medium as described previously. The cultures underwent two washes with 0.9% NaCl followed by centrifugation at 10 000 rpm for 2 min, and OD_600_ was adjusted to 0.1. For the first layer, 10 ml of 0.1× TSA (1.5% agar) were poured into petri dishes and allowed to dry for 30 min. Then, 100 μL of each target bacterium was added to 10 ml of 0.1× TSB containing 0.9% agar preheated to 45°C. This mixture was evenly spread on top of the 0.1× TSA and dried for an additional 30 min. Subsequently, 5 μL of each *B. subtilis* suspension was spotted on each plate. The plates were then incubated at room temperature, followed by examination of the inhibition zones on the lawn formed in the top layer.

Similarly, we investigated the impact of exometabolites produced by SynCom members on the growth properties of *B. subtilis strains*. Spent media from SynCom cultures were collected after 48 h of growth in 0.1× TSB at 25°C and 250 rpm, filtered through 0.22 μm filters, and stored at 4°C. Growth curves were generated in 96-well microtiter plates. Each well contained 180 μL of 0.1× TSB supplemented with 5% spent media from each SynCom strain and 20 μL of either *B. subtilis* WT or its mutants. Control wells contained only 0.1× TSB medium without spent media supplementation. Cultivation was carried out in a Synergy XHT multi-mode reader at 25°C with linear continuous shaking (3 mm), monitoring optical density at 600 nm every 5 min.

### Competition assay

Overnight cultures of the SynCom members and the *gfp-*labeled *B. subtilis* (WT; *sfp* and *srfAC*) were pelleted (8000 rpm, 2 min) and resuspend in 0.1× TSB at an OD_600_ of 0.1. Next, 200 μL of a SynCom member was inoculated in the first row of a 96-well microtiter plate. From there, the SynCom member was 10-fold diluted by transferring 20 μL of culture to the next row containing 180 μL of medium. This process was repeated for 6 dilution steps. Subsequently, 20 μL of the GFP-labelled *B. subtilis* variants was added to each well to establish the co-culture. Monocultures of both the SynCom member and *B. subtilis* variants served as controls to calculate competitiveness in co-culture. Cultivation was carried out in a Synergy XHT multi-mode reader (Biotek Instruments, Winooski, VT, US), at 25°C with linear continuous shaking (3 mm), monitoring the optical density and GFP fluorescence (Ex: 482/20; Em:528/20; Gain: 35) every 5 min. Kinetic parameters were estimated using the package GrowthCurver [50] in R.

### *Bacillus subtilis* specialized metabolites induction by the synthetic community spent media

The WT strain was inoculated in the presence of culture spent media from the SynCom members. The spent media were obtained after 48 h of growth in 0.1× TSB and filtered through at 0.22 μm. 10% of the spent media to Erlenmeyer flasks containing potato dextrose broth (15 ml in 100 ml flasks), followed by inoculation with an overnight culture of P5_B1 (OD_600_ = 0.1). After 48 h of incubation at 25°C and 220 rpm, the cultures were centrifuged, filtered, and subjected to HPLC analysis for surfactin detection. Surfactin was detected already at 0.1 μg/ml using a purified standard.

### Assessment of *Bacillus subtilis* establishment in diverse synthetic communities

To elucidate the role of surfactin in determining the establishment of *B. subtilis* within synthetic communities, we investigated whether P5_B1 can establish in various SynComs in a surfactin-dependent manner, using a methodology like the one described above for the competition assay. For this purpose, we selected five previously characterized bacterial SynComs, each with distinct compositions in terms of taxonomy and number of members, assembled for various objectives (Table S1). In all cases, the SynCom members and the *gfp*-labeled *B. subtilis* strains (WT and *srfAC*) were cultured overnight in 0.5× TSB. Following two washes with 0.9% NaCl, the ODs were adjusted to 0.1 in 0.1× TSB. The SynCom members were mixed in a 1:1 ratio and then inoculated and diluted in a 96-well plate. Subsequently, 20 μL of the *gfp*-labeled *B. subtilis* variants were added to each well to create the co-culture (Fig. S1). Monocultures of both the SynCom member and *B. subtilis* variants were included as controls to determine competitiveness in the co-culture. Cultivation conditions and data analysis were conducted as described for the competition assay. Each experiment was performed with at least three independent replicates per treatment.

### Statistical analysis

Data analysis and graphical representation were performed using R 4.1.0 [51] and the package ggplot2 [52]. Statistical differences in experiments with two groups were explored via Student’s *t-*tests. For multiple comparisons (more than two treatments), one-way analysis of variance (ANOVA) and Tukey’s honestly significant difference (HSD) were performed. In all the cases, normality and equal variance were assessed using the Shapiro–Wilks and Levene test, respectively. Statistical significance (α) was set at 0.05. Detailed statistical analysis description for each experiment is provided in figure legends.

## Results

### Description of the artificial soil system inoculated with synthetic community

**Figure 1:**
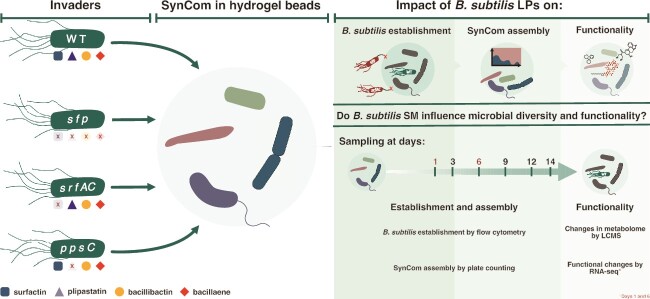
A schematic diagram illustrating the experimental design, and the research question of the main experiments conducted. *B. subtilis* P5_B1 and its NRP-deficient mutants were inoculated into a 4-member SynCom propagated in the hydrogel bead microcosms.

To assess the role of *B. subtilis* SMs in shaping bacterial community assembly under soil-like conditions, we previously customized a hydrogel matrix that supports the axenic growth of multiple bacterial strains and enables the quantification of specific *B. subtilis* LPs (i.e. surfactin and plipastatin) [42]. We subsequently assembled a four-membered bacterial SynCom obtained from the same sample site as *B. subtilis* P5_B1 [53]. We selected these four isolates due to their shared origin with P5_B1, their stable co-existence in our hydrogel beads system, and their morphological distinctness, which allowed for straightforward quantification by plate count at detection limits around 10^2^ CFU/g of beads. Although the relative abundance of each of the four strains fluctuated throughout the experiments, all four members were still detectable for up to three days of sampling (Fig. S2A). At the end of the experiment, we observed a clear strain co-existence pattern in the SynCom as previously reported: *Stenotrophomonas indicatrix* and *Chryseobacterium* sp. were the most dominant strains, *R. globerulus* was kept at low density whereas *Pedobacter* sp. was below our detection limit after day 3 (Fig. S2B). Using this established experimental system, we explored the role of LPs in the successful establishment of *B. subtilis*, as well as in SynCom assembly and functionality. A schematic diagram illustrating the core experimental design, and the scientific questions is presented in Fig. 1.

### Surfactin production facilitates *Bacillus subtilis* P5_B1 establishment in a four-member synthetic community

**Figure 2:**
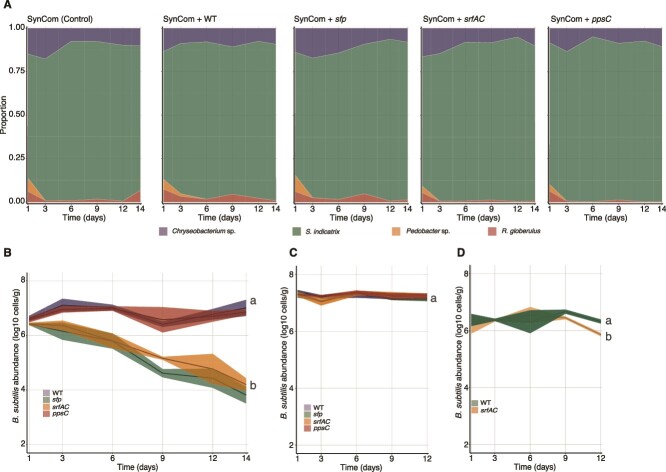
Surfactin production facilitates *B. subtilis* establishment in a SynCom but does not alter its composition in the soil-like environment over time. A set of *gfp*-labeled *B. subtilis* strains (Surfactin producers: WT and *ppsC*, non-producers: *sfp* and *srfAC*) were introduced into the SynCom, and their populations were followed over time to determine the role of LPs in SynCom assembly and their contribution to *B. subtilis* establishment in the simplified system. (A). SynCom assembly after the different *B. subtilis* variants were introduced. Members abundances are represented as the proportion occupied by each member relative to the total biomass in the system. (B) *B. subtilis gfp*-labeled population dynamics after introduction in the SynCom. Surfactin producers (WT and *ppsC*) population size remains stable over time whereas non-producers (*sfp* and *srfAC*) sharply decline by the end of the experiments suggesting that surfactin production is crucial for *B. subtilis* establishment in the SynCom. (C) *B. subtilis* WT and its derivates mutant growth dynamics when propagated individually in the hydrogel microcosms. (D) Complementation assay. A mixed population (1:1) of the WT and the *srfAC* was propagated in the hydrogel beads in the presence of the SynCom. The presence of the WT strain (surfactin producer) rescued the surfactin-deficient mutant. The letters represent significant differences among groups at day 14 (one-way ANOVA and Tukey honest tests). The experiment was conducted independently twice with *n* = 3 in both cases. The data were pooled and analyzed together.

To evaluate the contribution of specific LPs to P5_B1 establishment in the SynCom, we co-cultivated either the WT strain or the SM production-impaired mutants (*sfp*, *srfAC* and *ppsC*) in the presence of the SynCom using the hydrogel matrix that mimics soil characteristics [41, 42]. Initially, we confirmed that P5_B1 and its mutant derivatives grew and produced the expected LPs when cultivated axenically in the soil-like system. All *B. subtilis* strains colonized the hydrogel system at comparable rates (ANOVA at day 14, *P =* .87), demonstrating a similar population dynamic pattern: a one-log increase within a day followed by a plateau of nearly 1x10^7^ CFU/g of the hydrogel after three days of cultivation, which was maintained up to the final sampling time on day 14 (Fig. 2C).

When introduced to the SynCom, the WT and *ppsC* mutant (that produce surfactin but not plipastatin), successfully colonized the beads and maintained their population at approximately 1x10^7^ CFU/g throughout the experiment, comparable to the titers obtained in axenic cultivation. In contrast, the population size of the *B. subtilis* genotypic variants impaired in non-ribosomal peptides (*sfp*) or solely in surfactin (*srfAC*) production sharply declined during the first six days. By the end of the experiment, the cell titers decreased to around three log-fold below the initial population levels (ANOVA, *P* < .01) (Fig. 2B). Following up on these observations, we investigated whether the WT strain could rescue the *srfAC* mutant by co-inoculating a mixture of both strains into the SynCom. In this co-culture, the WT strain remained more competitive than the *srfAC* mutant. However, the presence of the WT strain, and presumable its surfactin production capability, evidently rescued the *srfAC* mutant, as its decline was less pronounced compared to when introduced alone into the SynCom (Fig. 2D).

**Figure 3:**
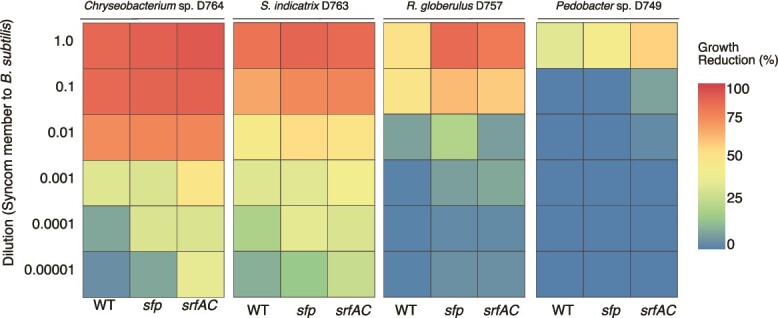
Impact of individual SynCom members on *B. subtilis* growth. A set of *B. subtilis* strains were co-cultured with each SynCom member at different ratios. The GFP signal was used as a proxy for the respective *B. subtilis* strains’ growth in co-culture and the area under the curve of growth was used as culture productivity parameter. The impact of being co-culture with each SynCom member was estimated as % growth reduction = [(Growth^Bsub Monoculture^ - Growth^Bsub Co-culture^)/Growth^Bsbu Monoculture^)] × 100.

Subsequently, we investigated the potential contribution of individual SynCom members to the decline of the surfactin-deficient strains using a *pair-wise* competition assay in planktonic cultures. Here, varying ratios of each SynCom member and *B. subtilis* were assessed and the reduction of the growth (i.e. area under the curve) relative to the monoculture was measured. *B. subtilis* populations experienced a significant reduction when co-cultured with *S. indicatrix* D763 and *Chryseobacterium* sp. D764 at the highest ratio (1, 0.1, 0.01 of the tested strain relative to the *B. subtilis* cultures), irrespective of *B. subtilis* capability to produce surfactin. However, in co-cultures where the SynCom members were diluted (more than 0.01 relative to *B. subtilis*), *B. subtilis* strains lacking surfactin production were outcompeted by *S. indicatrix* D763 and *Chryseobacterium* sp. D764. Overall, *B. subtilis* WT showed greater competitiveness against these SynCom members, maintaining higher growth at higher dilution ratios compared to the *sfp* and *srfAC* mutants. In contrast, the less competitive strains in the bead systems, *R. globerulus* D757 and *Pedobacter* sp. D749, only impacted *B. subtilis* growth at the highest co-culture ratio, with strains lacking surfactin production exhibiting comparable growth to WT (Fig. 3).

### *Bacillus subtilis* secondary metabolites do not have a major impact on synthetic community assembly

**Figure 4:**
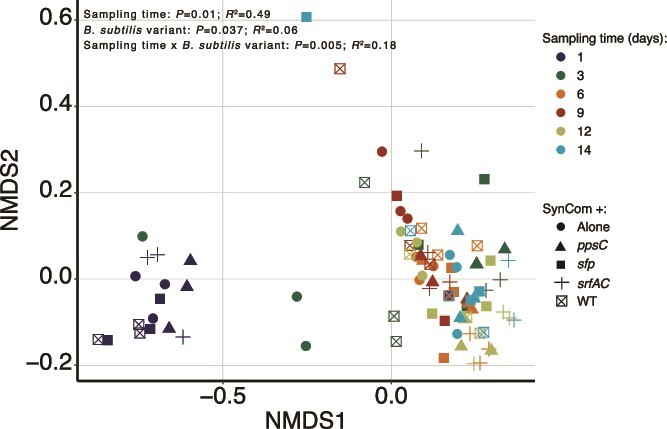
Changes in SynCom composition upon *B. subtilis* inoculation. Bray–Curtis distance NMDS ordination plot performed on the CFU data of the SynCom after *B. subtilis* introduction for comparing the effects of sampling time (colors) and the variant of *B. subtilis* (shapes) on SynCom composition. The multivariate analysis of SynCom composition was performed using a PERMANOVA on Bray–Curtis dissimilarity matrix obtained from the CFU counts dataset using the function adonis2 (R package vegan). The model was adjusted as: Y ~ time:variant. The *P* values and *R^2^* are reported as an inset within the figure.

Motivated by our observation that SM production, specifically surfactin, plays a crucial role in *B. subtilis* establishment success, we investigated if these SMs impact the SynCom composition over time. To do this, we evaluated the abundance of SynCom members (CFU) using NMDS and PERMANOVA (Fig. 4). Regardless of the *B. subtilis* strain introduced, the SynCom followed similar assembly dynamics as we described above: *S. indicatrix* and *Chryseobacterium* sp. dominated the community whereas *R. globerulus* and *Pedobacter* sp. were less abundant (Fig. 2A and Fig. S3). Estimation of the growth rates and the carrying capacity of each SynCom member in 0.1× TSB revealed that *S. indicatrix*, the most dominant strain, grew significantly faster and reached the highest cell density whereas *Pedobacter* sp. grew at the slowest rate (Fig. S4). This could explain the observed SynCom composition on the hydrogel system, which was dominated by the fastest growers and more productive strains.

A fixed-effect PERMANOVA using sampling time, *B. subtilis* variants and their interaction (how sampling time and *B. subtilis* variants jointly influence community composition) confirmed that the main driver of SynCom composition was the sampling time (PERMANOVA, *R^2^* = 0.49, *P* = .001), with a minor effect of *B. subtilis* strain introduced (PERMANOVA, *R^2^* = 0.06, *P* = .037) and the interaction (PERMANOVA, *R^2^* = 0.18, *P* = .005). Overall, the results suggested that introducing either the WT or its SM-impaired mutants did not have a major impact on the SynCom assembly, with the differences mainly explained by the sampling time (Fig. 2A and Fig. 4).

We investigated whether the antagonistic activity between the SynCom members and *B. subtilis* could explain our observations. Using an *in vitro* inhibition test, we found that the less competitive strains, *Pedobacter* sp. D749 and *R. globerulus* D757, were both susceptible to *B. subtilis*. Specifically, the antagonistic activity against *Pedobacter* sp. D749 was linked to NRP production, particularly surfactin, whereas *R. globerulus* was inhibited by all the variants. This suggests that other classes of SMs beyond NRP, produced by *B. subtilis*, may contribute to the inhibition of these two species. Nevertheless, the SynCom-abundant strains, *S. indicatrix* D763 and *Chryseobacterium* sp. D764, displayed no growth reduction by *B. subtilis* and its SMs, as evidenced by the absence of inhibition halos (Fig. S5).

### *Bacillus subtilis* and synthetic community metabolome are both altered during the establishment experiments

**Figure 5:**
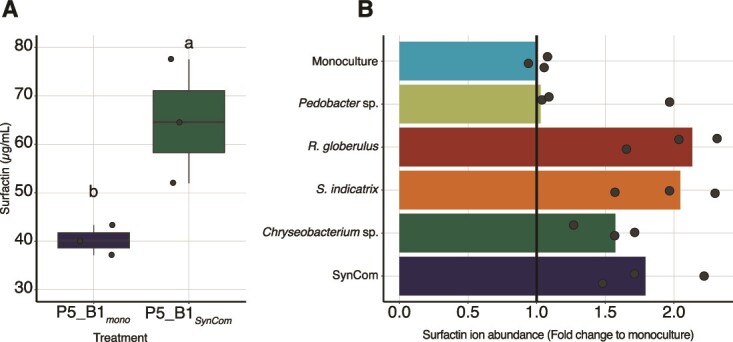
The full SynCom and individual SynCom members induce surfactin production both in the bead system and in liquid culture. (A) Surfactin production in the bead system by P5_B1 increased when co-cultivated with the full SynCom compared to pure *B. subtilis* monoculture. The concentration was estimated from the last day of sampling in the *B. subtilis*, SynCom co-cultivation experiment. (B) Changes in surfactin production when P5_B1 was supplemented with cell-free supernatants obtained from individual SynCom members and the full SynCom in liquid cultures. In both experiments surfactin production was quantified by UHPLC and three replicates (*n* = 3) were performed per treatment.

To explore the role of *B. subtilis* secondary metabolites in shaping the SynCom metabolome and how surfactin production was modulated in co-cultivation, we profiled both the SynCom and *B. subtilis* metabolome at day 14 of the experiment using liquid chromatography-mass spectrometry (LC–MS). A targeted approach revealed that the production of surfactin was significantly increased when the WT was grown in the presence of the SynCom compared with the WT production in axenic cultures (*t*-test, *P* = .0317) (Fig. 5A). This finding was further validated *in vitro* by supplementing P5_B1 cultures with cell-free supernatants from each of the SynCom members or all strains together. Here, the spent media from both the monocultures and the SynCom induced surfactin production, with the highest increase observed when P5_B1 was supplemented with *R. globerulus* supernatant (Fig. 5B).

**Figure 6:**
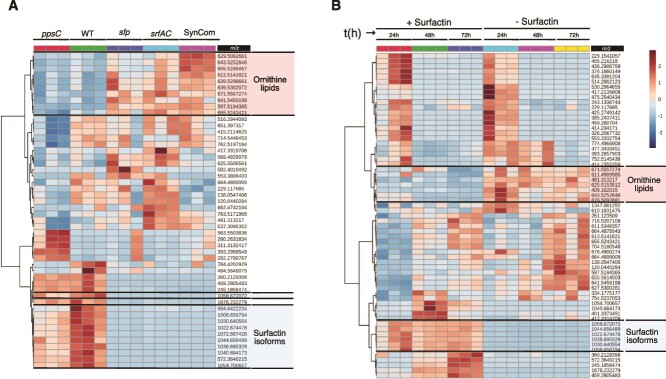
Untargeted metabolic analysis revealed extensive metabolic changes in the co-cultivation experiments. (A) A heatmap visualization based on the significantly increased or decreased chemical features (m/z) in the co-cultivation experiments. A set of chemical features (m/z), related to OLS were strongly modulated in presence of surfactin producer (WT and *pps*C) (Upper brackets). Similarly, m/z associated with surfactin isoforms were detected in the system (Bottom brackets). (B) Changes in the SynCom chemical features when supplemented with pure surfactin. The visualization shows the variation over the time (24, 48, and 72 h) of the main chemical features produced by the SynCom. Similarly, the same group, OLS-lipids, were produced less in presence of pure surfactin (upper brackets). The heatmaps were made on the feature abundances retrieved from the ESI-MS chromatogram.

Although most of the molecular features (*m/z*) detected in our system remained unidentified, the molecular network clearly shows the presence of the *B. subtilis* LPs, plipastatin, and surfactin, and their analogs. Moreover, the presence of ornithine lipids (OLs) was observed in the dataset [54]. These metabolites are derived from Gram-negative bacterial cell outer membrane as surrogates of phospholipids under phosphate-limited conditions [55] (Fig. S6). The lipid abundances (*m/z* between 597 and 671) increased in the SynCom alone, indicating this conversion of phospholipids to OLs occurs in the absence of *B. subtilis.* Ecologically, OLs have been linked to stress response [54]. When surfactin producers (WT or *ppsC* mutant) were introduced into the system, the presence of OLs was strongly reduced. In contrast, with the *sfp* and *srfAC* mutants, OLs remained at levels comparable to the SynCom alone (Fig. 6A). We corroborated this observation by conducting an experiment with the SynCom in the presence of pure surfactin. Here, the same group of compounds (*m/z* features) was altered in the surfactin-supplemented SynCom culture as in the presence of surfactin-producing *B. subtilis* co-cultures, although these were abundant in the control samples (i.e., without *B. subtilis*) (Fig. 6B).

### Less competitive strains of the synthetic community were the most transcriptionally affected species by *Bacillus subtilis* specialized metabolites

To dissect the mechanism of how surfactin facilitates *B. subtilis* establishment within the SynCom, a meta-transcriptomic approach was conducted comparing the transcriptional profile of the SynCom challenged with the WT and the *sfp* mutant. In total, 430 genes and 490 genes were differentially expressed (DEG) in the SynCom after 1 and 5 days, respectively, inoculated with the WT compared with the sample seeded with the *sfp* mutant. In both sampling days, the less competitive strains, *Pedobacter* sp. D749 and *R. globerulus* D757 had the highest number of differentially expressed genes (DGEs) in the system, accounting for around the 83% of DEGs at day 1 and 95% of those at the last sampling point (Fig. S7). Subsequently, we explored the distribution of clusters of orthologous groups (COG categories) among the DEGs genes to discover which processes within the SynCom are potentially affected by the introduction of either the WT or *sfp* mutant. Here, many DEGs were not annotated or classified as COG S, an unknown function. However, cell wall/membrane/envelope biogenesis (COG M) and amino acid transport and metabolism (COG E) were the most abundant functional categories among the genes downregulated in the SynCom with WT strain added relative to the SynCom in the presence of *sfp* mutant (Fig. S8).

We explored the functions and enrichment pathways of DEGs for the less competitive strains (*Pedobacter* sp. D749 and *R. globerulus* D757). The GO enrichment analysis revealed that both strains responded transcriptionally differently in the presence of the WT strains. Whereas the enriched biological processes in *R. globerulus* D757 were related to defense mechanisms or response to other organisms, upregulated processes in *Pedobacter* sp. were linked to amino acid transport, specifically histidine (Fig. S9).

### Surfactin-facilitated establishment of *Bacillus subtilis* is conserved across diverse synthetic communities

**Figure 7:**
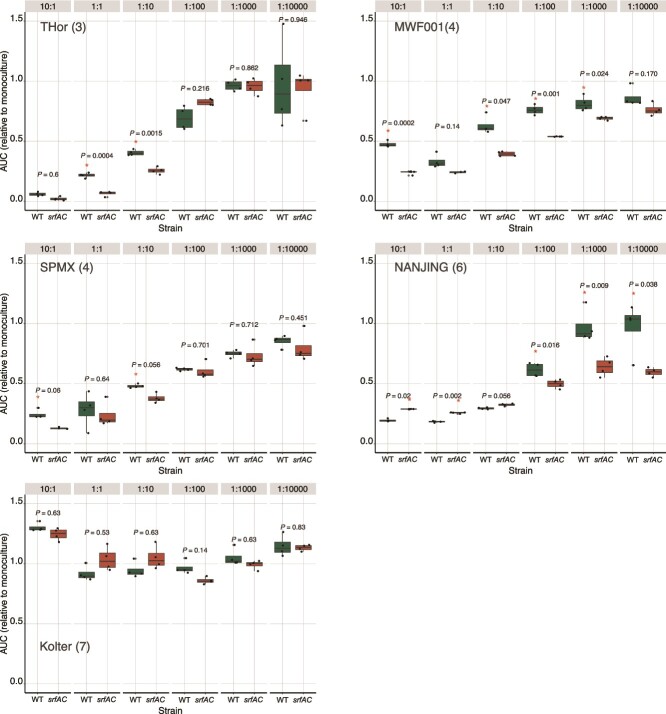
*B. subtilis* P5_B1 establishment in different publicly available SynComs. The WT and its derivate mutant impaired in surfactin production (*srfAC*) were co-cultured with different SynCom at decreasing ratios (see Methods and SI for details). The *gfp* signal was used as proxy for *Bacillus* establishment in the co-cultures during 24 h incubation period. The area under the curve (AUC) ratio (*B. subtilis* growth in co-culture in each SynCom dilution to *B. subtilis* growth in monoculture) was used as the *B. subtilis* establishment parameter. “^*^” indicates that the value of the AUC ratio was significant and its position denotated which strain had a higher value. “ns” indicates no significance (*P* < .05, Student’s *t*-test adjusted for multiple testing by the Benjamini–Hochberg method). The name inset the plot indicates the lab or the name of the SynCom, in parenthesis the number of members composing the community.

To survey if surfactin is important for establishment of *B. subtilis* P5_B1 within diverse microbial communities, we assessed the abundance of WT and surfactin-deficient mutant in five previously published and characterized SynComs [35, 56–61]. These SynComs varied in composition, reflecting different functionalities and ecological niches. Overall, the co-culture experiments revealed that the ability of *B. subtilis* to establish within the SynComs depended on surfactin production, SynCom composition (number of members), and the inoculation ratio. In most SynComs, except for the Kolter Lab’s SynCom which was broadly invaded, both the WT and the *srfAC* mutant displayed reduced growth at a high inoculation ratio of SynCom (10:1, 1:1, 1:10). However, the WT, which produces surfactin, generally reached higher population densities compared to the surfactin-deficient mutant across most SynComs. Although the difference between the WT and *srfAC* mutant was less pronounced in these shaken cultures compared with the tests performed on the alginate bead microcosm, this could be due to the lack of spatial structure present in the surface-attached communities or the differences in oxygen diffusion between the two experimental setups. When *B. subtilis* was inoculated at high ratios relative to the SynComs, the growth dynamics resembled those observed in axenic cultures of both the WT and *srfAC* mutant (Fig. 7).

## Discussion

Secondary metabolites have traditionally been studied for their antimicrobial or anticancer properties. However, several of these natural products exert multifaceted functions, influencing the physiology of the producing microorganism and modulating interactions with other organisms [26, 28]. Understanding the role of these compounds in natural habitats (*e. g.* in soil) is crucial for optimizing their use and biotechnological applications. However, this has been challenging due to the chemical and biological complexity and the limitations of quantifying SMs *in situ*. Therefore, this study aimed to elucidate the contribution of cyclic LPs, particularly surfactin and plipastatin, in the establishment and functional dynamics of both *B. subtilis* and SynCom members in a soil-mimicking environment. Our key findings demonstrate that surfactin production facilitates the establishment success of *B. subtilis* across multiple SynComs. Whereas surfactin was crucial for *B. subtilis* competitiveness, its production did not markedly alter the overall composition of the SynCom. Additionally, the metabolomic and transcriptomic analysis revealed that surfactin modulates both the producer and SynCom metabolic landscapes. Together, our results support past observations and the long-standing hypothesis, that bacteria lacking secondary metabolite production are less competitive than SM-producing wild-types [6, 62, 63].

We experimentally demonstrated the contribution of surfactin in *B. subtilis* success when inoculated in the presence of a SynCom using a reductionist approach: four-member bacterial SynCom propagated in microcosms based on an artificial hydrogel matrix [42, 53]. One of the biggest methodological challenges in studying SM-driven microbial interactions is to mimic the environmental conditions. Consequently, the need for developing model systems of intermediate complexity for elucidating the ecological role of these molecules and shedding light on microbiome assembly-related questions has been widely stated [30, 32, 64–66]. This is because classic axenic *in vitro* assays do not resemble crucial aspects of microbial niches, whereas natural samples are far too complex to dissect the underlying processes at the molecular level. Our SynCom is not intended to represent the natural sample site, i.e. Dyrehaven soil community, where all strains used in this study were isolated, but rather, it represents a reproducible, trackable, and easy-to-set bacterial assemblage useful for testing the role of SMs in SynCom assembly, and together with the soil-mimicking matrix, might help to overcome the bottlenecks imposed by soil complexity in terms of microbial diversity and SMs quantification. The described system aligns conceptually with recent approaches that used transparent microcosms mimicking the complexity of natural environments also allowing for testing hypotheses with statistical power in a controlled setup [41, 67–69].

Throughout the present work, we revealed the crucial role of surfactin in the establishment and persistence of *B. subtilis* within a set of diverse SynComs. Surfactin is by far one of the most-studied LPs and appears to confer a competitive advantage to *B. subtilis* under different conditions and environments. The relevance of this multifunctional SM has been demonstrated in biofilm formation [22, 70], swarming and sliding motility [71–73], root and phyllosphere colonization [74,75], and triggering induced systemic resistance (ISR) in plants [76,77]. Although it is not frequently highlighted as a primary function of surfactin, its contribution to the fitness of producers has been shown in different environmental conditions.

For instance, Luo et al. demonstrated that a *B. subtilis* strain impaired in surfactin production did not colonize rice sheaths inoculated with *Rhophitulus solani.* At the same time, WT increased its population size over time [78]. Similarly, Zeriouh and colleagues showed that *srfAB* mutant (of *Bacillus amyloliquefaciens* UMAF6614) presents reduced persistence in the melon phylloplane [79]. In soil, similar observations were made where surfactin-impaired mutants of *B. subtilis* were unable to colonize *Arabidopsis thaliana* roots [25,75]. In all these examples, the underlying mechanism links surfactin production with triggering *Bacillus* biofilm formation, surface spreading, and colonization.

Even though further experiments are needed to fully understand how surfactin enhance *B. subtilis* establishment in the SynComs, we hypothesize that surfactin-mediated niche colonization (spreading and biofilm formation) and alterations of the SynCom chemical landscape might play important roles in the observed phenomenon. *B. subtilis* P5_B1 is a strong biofilm producer both *in vitro* and on plant roots in laboratory settings [22]. We have shown here and previously [42] that P5_B1 produces surfactin in the microcosms at levels that are presumably required for timing of biofilm formation (~15 μg/ g of beads) [79], which may aid its attachment to the hydrogel beads, creating niches where *B. subtilis* could minimize competition for resources with other SynCom members. Furthermore, the surfactin-induced modulation of the overall SynCom chemical landscape (Fig. 6) could lead to niche differentiation. By reshaping community chemodiversity, surfactin may help to create distinct ecological niches. This differentiation could be essential for reducing competition and allowing the coexistence of the surfactin-producing strain within the community. Alternatively, surfactin production could help *B. subtilis* to cope with a potential oxygen depletion induced by the SynCom growth. Such function of surfactin has been recently demonstrated where surfactin production mediated *B. subtilis* survival via membrane depolarization and increased oxygen diffusion under low oxygen concentration [80].

We observe that the WT and the SM-mutant strains had hardly any influence on the composition and dynamics of the SynCom, but surfactin production altered the chemical diversity of the SynCom, besides the sensitivity of minor SynCom members to *B. subtilis* SMs. Several studies have highlighted that isolates of the *B. subtilis* species complex are not strong competitors of indigenous soil microbiota, and as a consequence, they did not shift the composition rhizosphere bacterial community to a considerable degree [81,82] or mainly influenced specific groups of the rhizospheres’ microbial community [83,84]. However, application of *B. subtilis* and its close-relative species in the rhizosphere improve plant health and resiliency, and SM production contributes to these properties.

Beyond the impact of the examined LPs on *B. subtilis* growth dynamics and SynCom composition, we found that surfactin production was stimulated in the presence of the SynCom or specific SynCom members compared to *B. subtilis* monocultures. This observation supports the well-established notion that microbial interactions play a crucial role in modulating the production of bioactive secondary metabolites [85–89]. Several studies have elegantly demonstrated the enhanced production of various natural products and their consequences for the producers (reviewed in [90]). For example, Andric *et al.* showed that *Bacillus velezensis*, a member of the *B. subtilis* complex, increases the production of bacillaene and surfactin upon sensing metabolic cues produced by *Pseudomonas sessilinigenes* CMR12a; leading to enhanced antibacterial activity by *B. velezensis* [91].

The increased surfactin production observed under our experimental conditions likely provides benefits to *B. subtilis* during community-level interactions. Beyond its antagonistic activity, particularly against closely related species, surfactin production is linked to multiple beneficial *Bacillus* phenotypes, potentially serving as defensive responses upon detecting bacterial competitors. For instance, phenotypes such as increased biofilm formation [70,92,93], enhanced motility [94], induction of sporulation [95], and secondary metabolite production [90,96] have been proposed as defensive mechanisms after sensing competitors [91,94,97]. However, the underlying mechanisms regulating *B. subtilis* SM production in response to their neighbor’s activity remain largely unknown. The so-called “competition sensing” hypothesis provides an ecological framework, suggesting that microbes have evolved the ability to sense hazard signals coupled with a stress response that enables a “counterpunch” by upregulating the production of antibiotics and toxins [29,98]. Similarly, the SynCom-secreted metabolome was modulated by the surfactin production. Here, we observed that primarily OLS lipids were downregulated when the SynCom was exposed to surfactin.

In sum, soil bacteria are well known for their potential to synthesize a plethora of SMs with a wide diversity of activities. Our understanding of the ecological roles of these metabolites under natural conditions has just begun to be unlocked. Our observations, gathered in an intermediate ecological complex experimental system revealed the role of surfactin in the ecology of the producers and how this SM impacts the metabolism of its interacting partners. Thus, we hypothesize that the production of multimodal secondary metabolites by *B. subtilis* is a refined strategy that contributes to fitness and persistence in natural habitats where competition could be thorough.

## Supplementary Material

LozanoAndrade_ISMEJ_SupplementaryInformation_R2_wraf013

Table_S1_wraf013

## Data Availability

Analysis scripts, raw and processed data have been deposited at Github (https://github.com/carlosneftaly/SurfactinSynCom_story). Raw sequence reads of the RNAseq campaign have been deposited at the Sequencing Read Archive (SRA) with BioProject ID PRJNA1145146. LC–MS data have been deposited at GNPS-MassIVE under MSV000094405.
