# Supplementary material for: Surfactin facilitates establishment of Bacillus subtilis in synthetic communities
Source: ISME J. 2025 Jan 23;19(1):wraf013. doi: 10.1093/ismejo/wraf013 (PMC11833321; doi:10.1093/ismejo/wraf013)
Supplement: LozanoAndrade_ISMEJ_SupplementaryInformation_R2_wraf013 [file lozanoandrade_ismej_supplementaryinformation_r2_wraf013.pdf]

Supplementary Figures

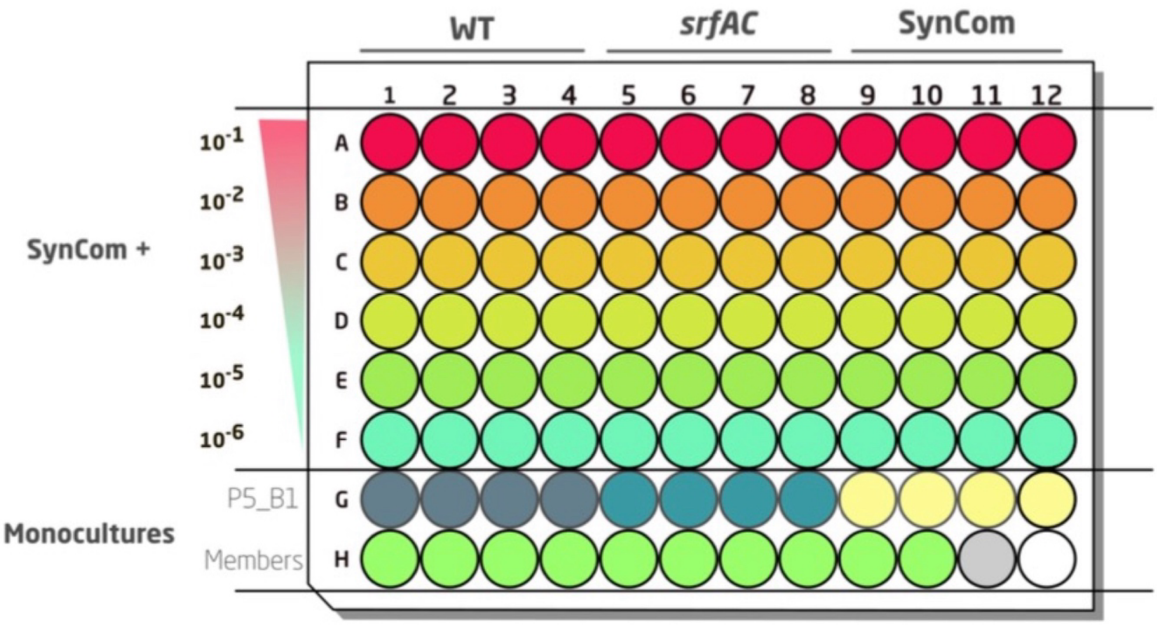

Fig S1. Microtiter plate setup for the invasion test. 200 µL of each SynCom were inoculated in the first row A. From that row, the SynCom members were 10-fold diluted by transferring 20 µL of culture to the next row containing 180 µL of medium. 6 dilution steps were made. Subsequently, 20 µL of *B. subtilis* the *gfp*-labelled variants were added to each well to conform to the co-culture. Rows G-H were dedicated to the two *Bacillus* strains and the individual members of each SynCom. Both *Bacillus* strains were cultured as a quadruplicate, and members were cultured as a quadruplicate when possible and a triplicate when not.

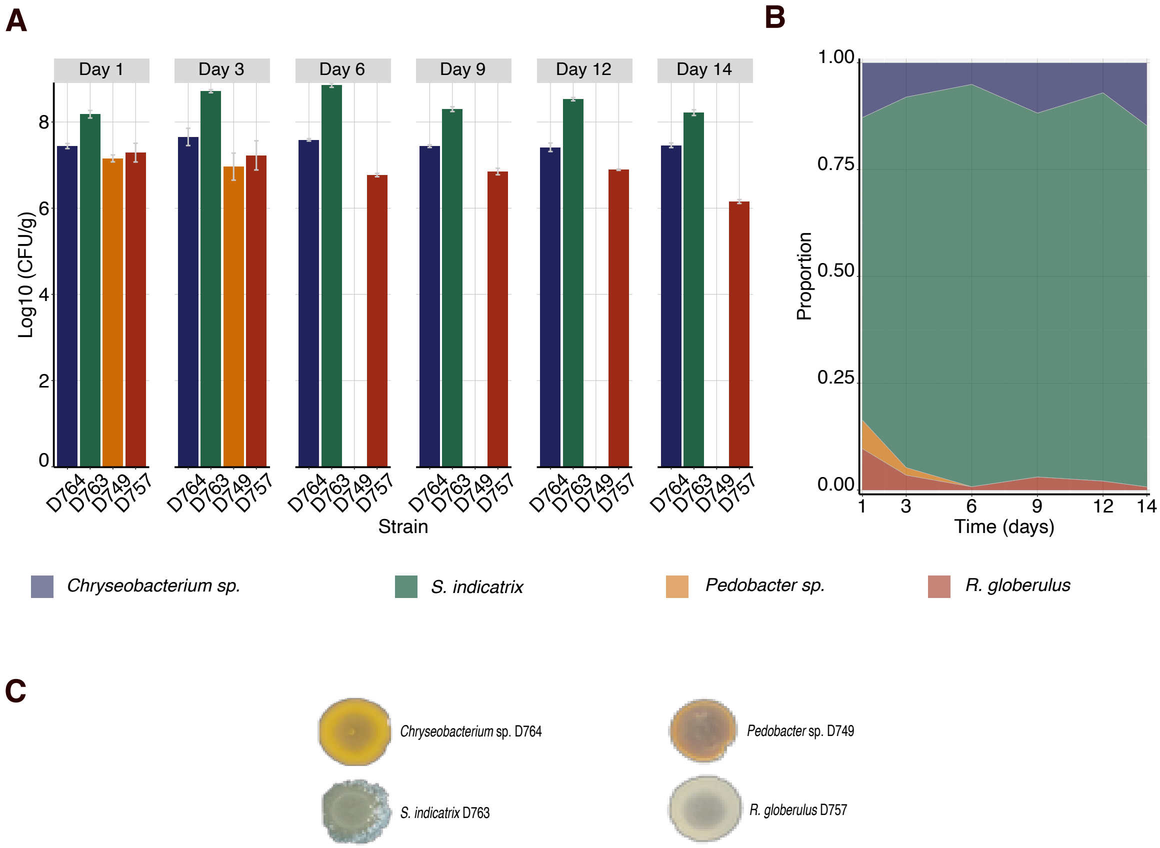

Fig S2. A stable, trackable, simplified bacterial synthetic community (SynCom) for studying chemical ecology. Overnight cultures of the four members were mixed and propagated in the hydrogel matrix. The abundance of each member was differentially determined as colony-forming units per gram of beads. (A). Population dynamics of each member over time. (B) Members abundances are represented as the proportion occupied by each member related to the total biomass in the system. (C) The colony morphology of the SynCom members allows us to differentially estimate their population in co-cultures. Error bars indicate standard deviation (n=3).

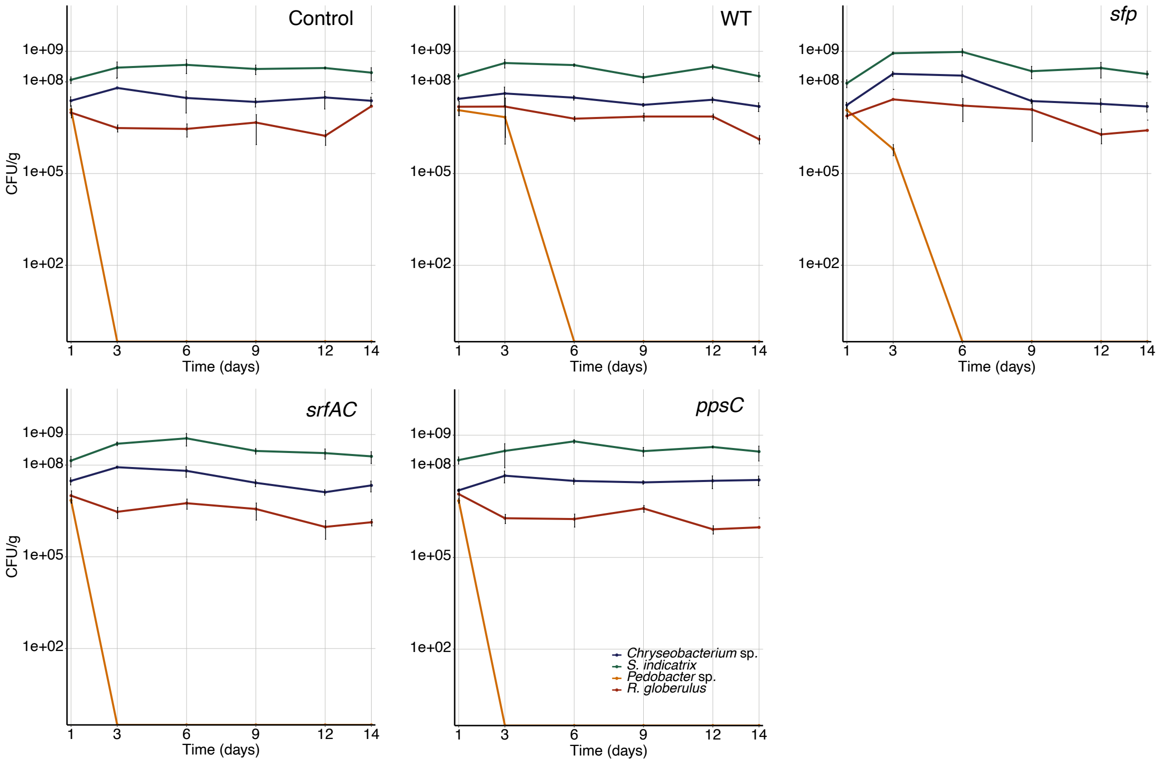

Fig S3. Raw counts (CFU/g) of each SynCom member when challenged with the different *B. subtilis* variants. These raw counts were used to generate the plot showing the SynCom composition over time as the proportion occupied by each member in Fig 2A. Each line represents a SynCom member and the point the mean and the standard deviation (n=3).

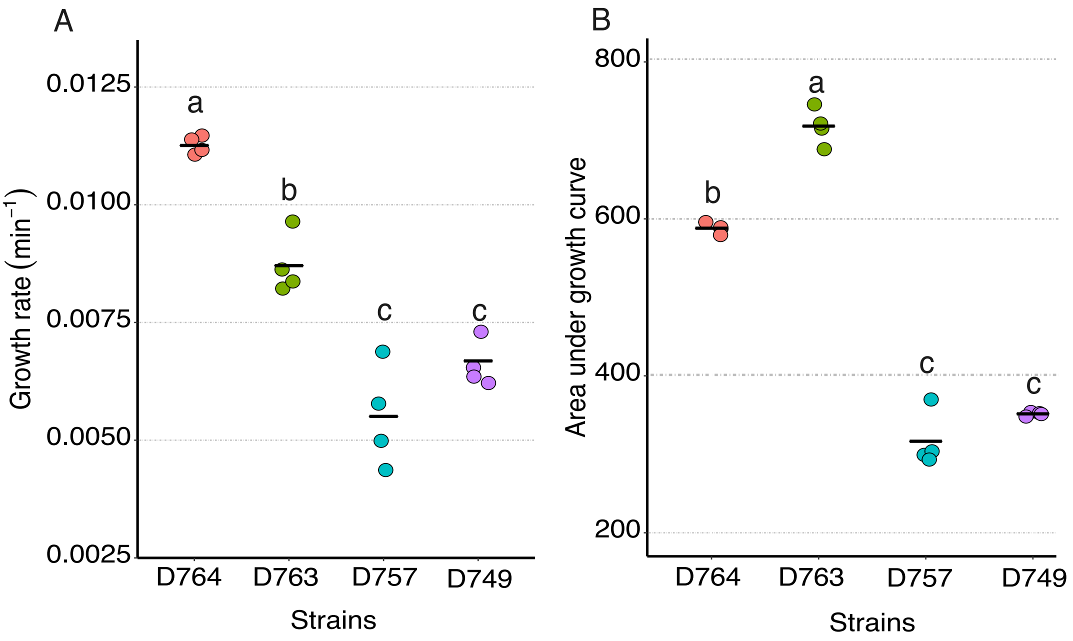

Fig S4. Growth properties of the SynCom members in liquid culture. The SynCom members were grown in 0.1× TSB at 25C and 225 rpm for 24 h. Then, the kinetics parameters were estimated using Growthcurver in R. *Chryseobacterium* sp. D764 and *S. inidcatrix* D763 are the fittest under these conditions as the growth rate and de AUDC revealed. (A) The growth rate of the SynCom members. (B) Area under growth curve as a measure of strain productivity at the end of the experiment. Data were derived from four replicates. Each point represents a sample, and the black line is the mean of the group. Significant differences were evaluated using ANOVA, and the letters indicate significant differences from a Tukey test (P<0.05).

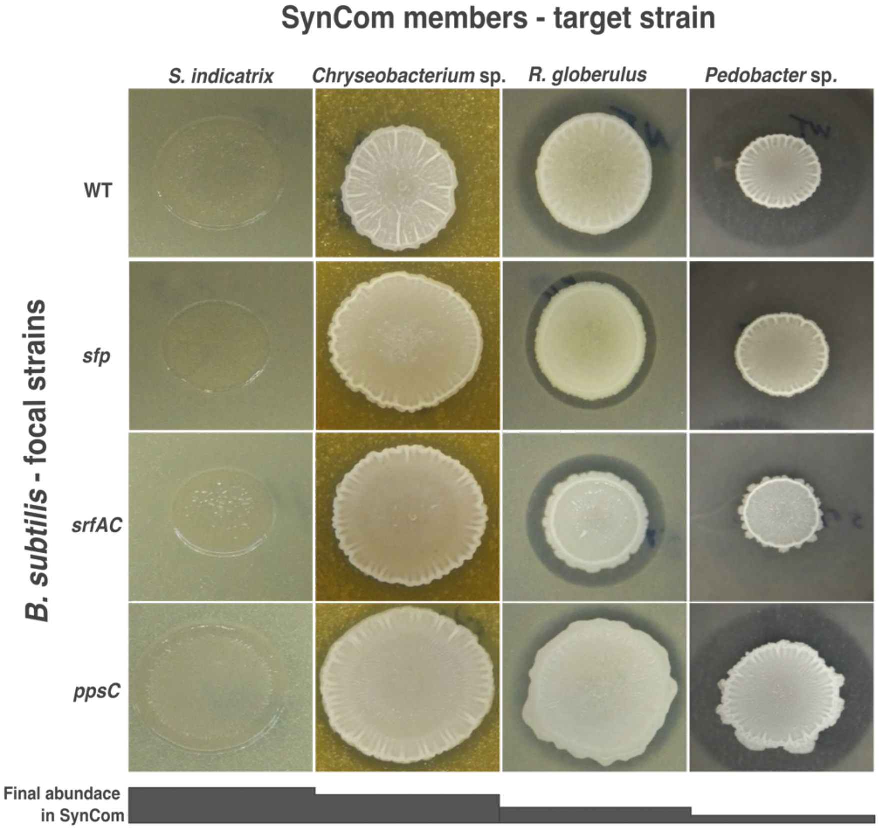

Fig S5. Antagonistic activity of *B. subtilis* strains against SynCom members. The SynCom members were inoculated in a soft layer of 0.1× TSA, forming a lawn. On top, 5µL of each *B. subtilis* strain was spotted. Then, the plates were incubated and inhibition halos were recorded. The appearance of a clear halo indicates antagonistic activity. *R. globerulus* was susceptible to *B. subtilis,* but such inhibition was not linked to NRPs production. In contrast, the inhibitory activity towards *Pedobacter* sp. was NRP-dependent, being surfactin crucial in such activity.

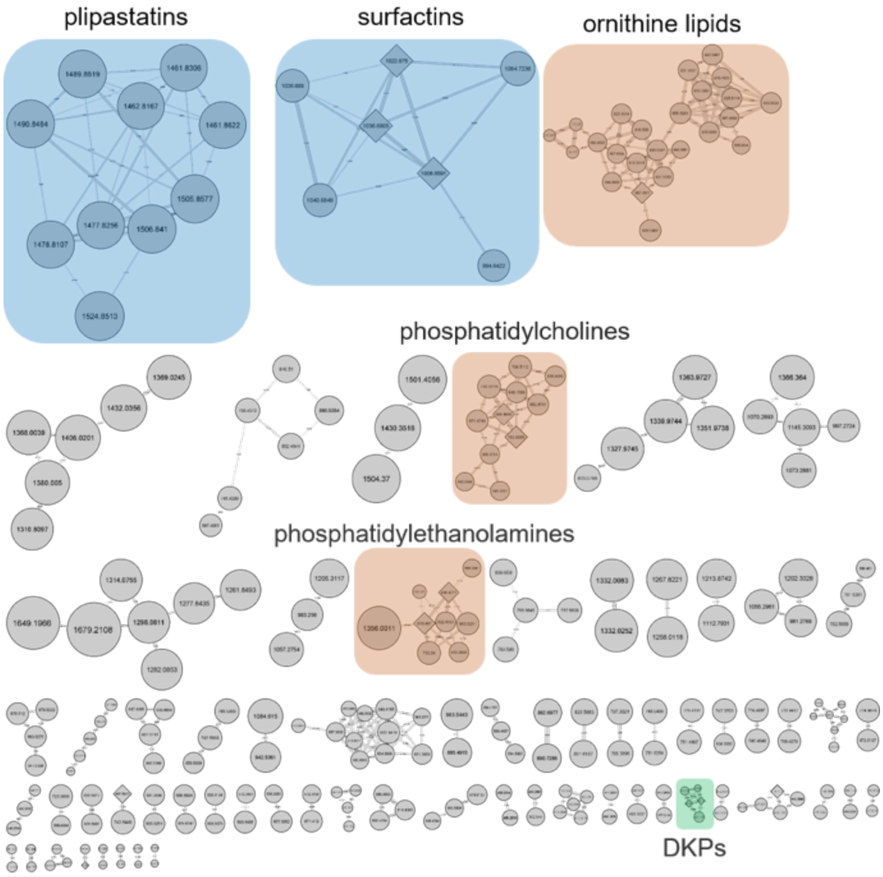

Fig S6. Feature-based molecular networking of the SynCom challenged with different variants of *B. subtilis*. Molecular networks of all detected features are presented in the plot. Identified chemical classes are highlighted on colors.

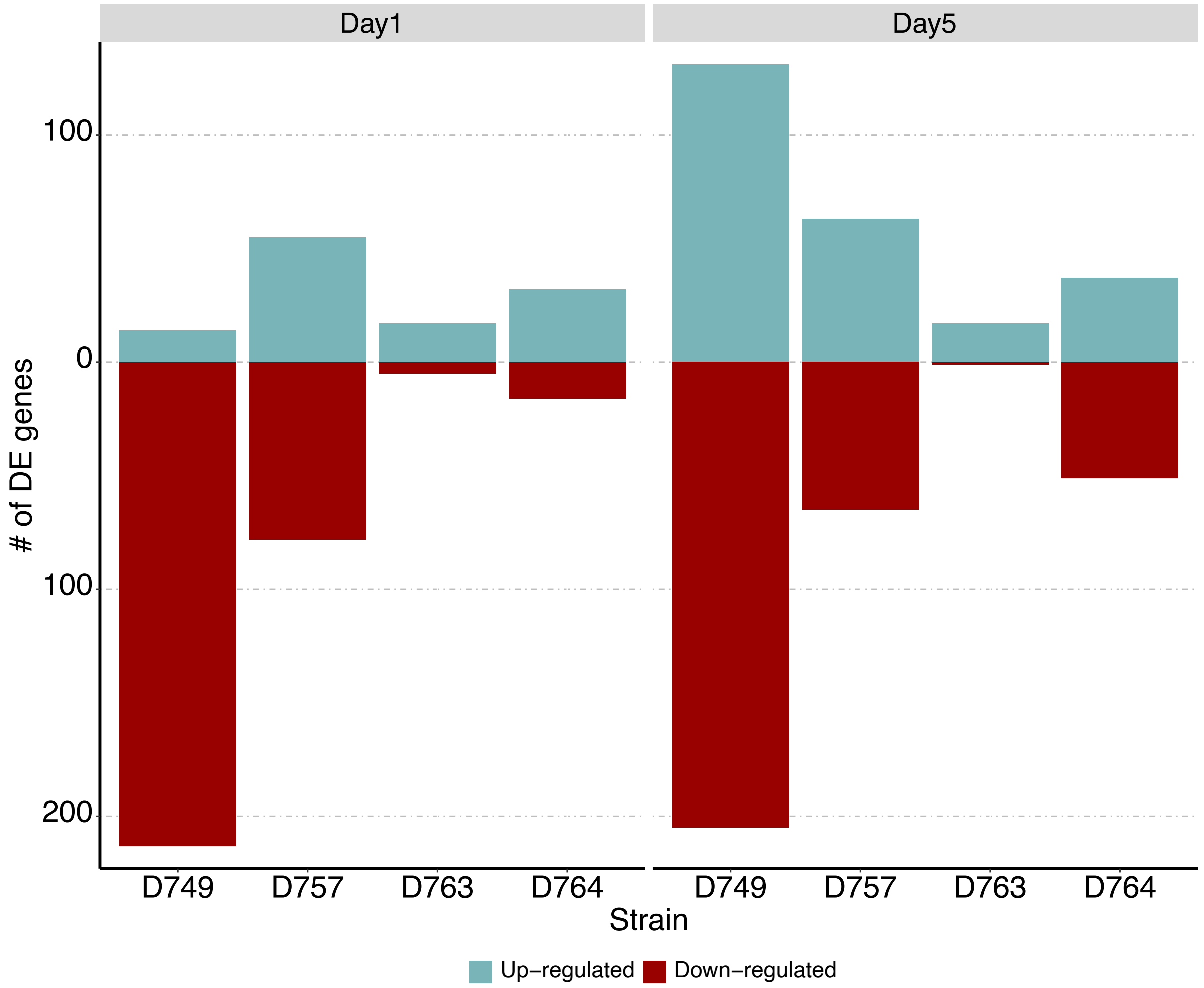

Fig S7. An overview of the differentially expressed genes of each strain over the time. Number of differentially regulated genes (log2FC ≥ |2| and p-value ≤ 0.01) in each species during co-cultivation experiment. In all the cases the comparisons were performed as WT vs. *sfp* mutant.

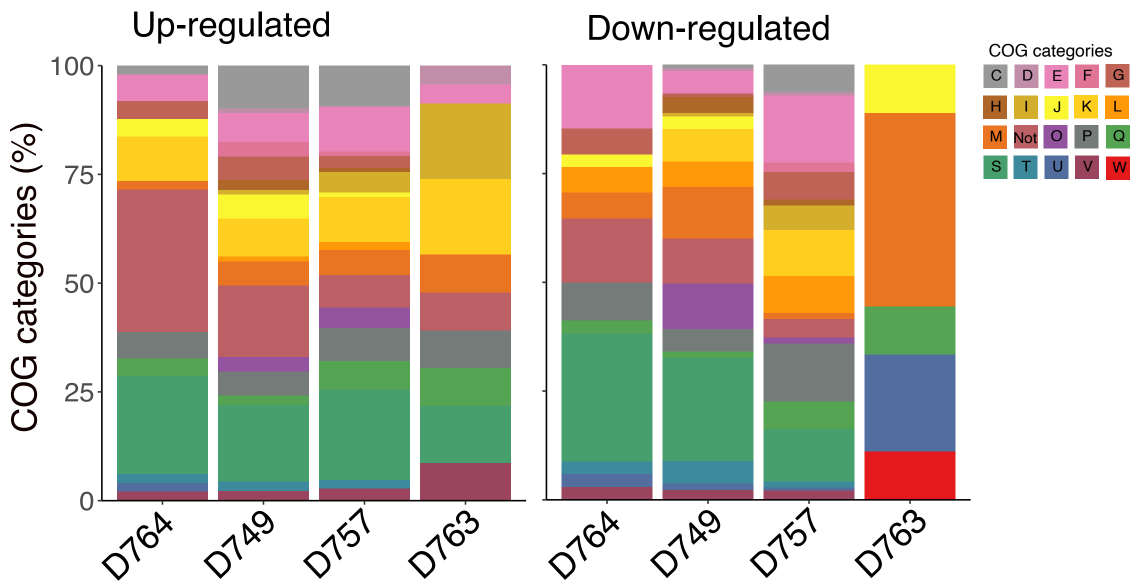

Fig S8. COG categories of genes up- or down-regulated by the four species in the SynCom challenged with the WT strain if compared to *sfp* ones at day 5 of sampling. The COG categories names can be accessed at the following link:
https://ecoliwiki.org/colipedia/index.php/Clusters_of_Orthologous_Groups_(COGs)

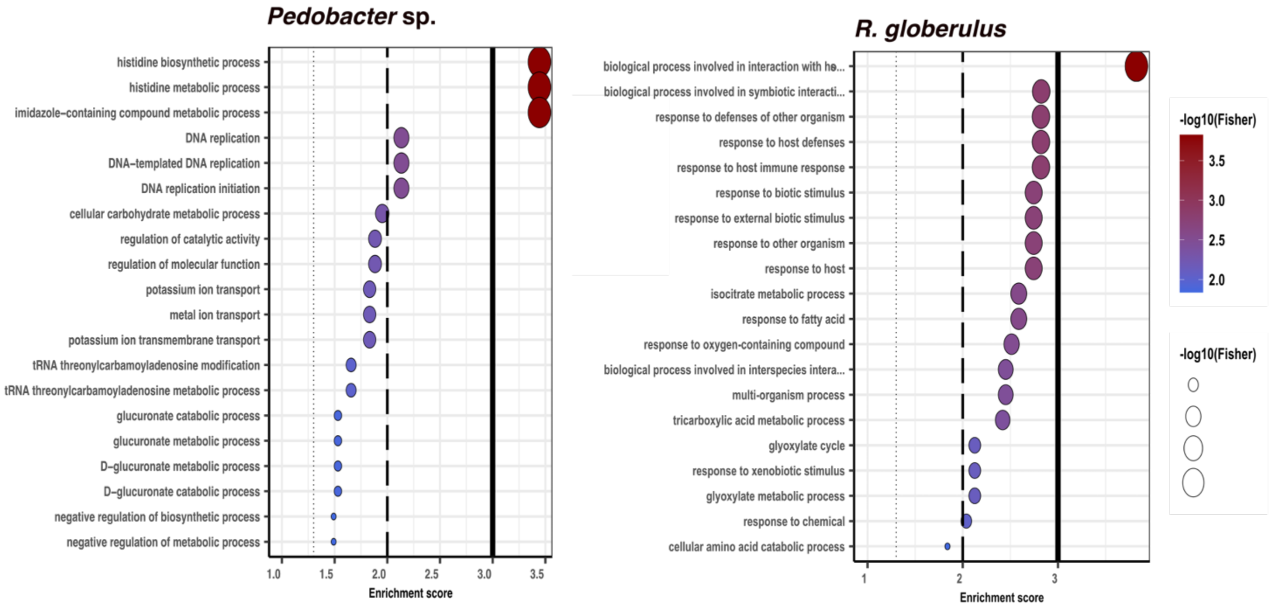

Fig S9. Biological processes in gene ontology (GO) enrichment analysis of DEGs during the *B. subtilis* – SynCom co-cultivation experiment. GO enrichment analysis was performed using topGO program in R. Only significantly enriched terms with corrected $P < 0.05$ were indicated. The color and size of each point represented the -log10 (FDR) values and enrichment scores. A higher -log10 (FDR) value and enrichment score indicated a greater degree of enrichment in SynCom co-cultivation experiment.
