# Supplementary material for: Surfactin facilitates establishment of Bacillus subtilis in synthetic communities
Source: ISME J. 2025 Jan 23;19(1):wraf013. doi: 10.1093/ismejo/wraf013 (PMC11833321; doi:10.1093/ismejo/wraf013)
Supplement: Table_S1_wraf013 [file table_s1_wraf013.pdf]

Table S1. List of strains used in this study

Strain	Description		Reference
P5_B1	*B. subtilis* natural soil isolate		[1]
DTUB38	P5_B1 *amyE*::$P_{hyperspank}$-*gfp* (Chl^{R})		[2]
DTUB186	P5_B1 *amyE*::$P_{hyperspank}$-*gfp* (Chl^{R}); *sfp*::Mls^{R}		
DTUB148	P5_B1 *amyE*::$P_{hyperspank}$-*gfp* (Chl^{R}); *srfAC*::Tn*10* ($Spec^{R}$)		
DTUB187	P5_B1 *amyE*::$P_{hyperspank}$-*gfp* (Chl^{R}); Δ*ppsC* (Tet^{R})		
DTUB188	P5_B1 *amyE*::$P_{hyperspank}$-*gfp* (Chl^{R}); Δ*pksL* (Chl^{R})		
	P5_B1 *amyE*::$P_{hyperspank}$-*mKate* ($Spec^{R}$)		
D749	*Pedobacter* sp.	CeMiSt SynCom	[3]
D757	*Rhodococcus globerulus*		
D763	*Stenothrophomonas indicatrix*		
D764	*Chryseobacterium* sp.		
Sr	*Stenotrophomonas rhizophila*	SPMX SynCom	[4]
Pa	*Paenibacillus amylolyticus*		
Mo	*Microbacterium oxydans*		
Xr	*Xanthomonas retroflexus*		
MWF001	*Agrobacterium tumefaciens*	MWF SynCom	[5,6]
	Comamonas testosteroni		
	Microbacterium saperdae		
	Ochrobactrum anthropi		
UW85	*Bacillus cereus*	Thor SynCom	[7]
UW101	*Flavobacterium johnsoniae*		
CI12	*Pseudomonas koreensis*		
XL380	*Acinetobacter baumanni*	Nanjing University SynCom	[8]
XL97	*Chryseobacterium rhizoplanae*		
XL95	*Enterobacter ludwigii*		
XL123	*Pantoea eucrina*		
XL272	*Pseudomonas stutzeri*		
WLL	*Comamonas odontotermitis*		
	Enterobacter cloacae	Kolter Lab SynCom	[9]
	Stenotrophomonas maltophilia		
	Ochrobactrum pituitosum		
	Herbaspirillum frisingense		
	Pseudomonas putida		
	Curtobacterium pusillum		
	Chryseobacterium indologene		

References

1. Kiesewalter, H. T. *et al.* Complete Genome Sequences of 13 *Bacillus subtilis* Soil Isolates for Studying Secondary Metabolite Diversity. *Microbiol. Resour. Announc.* **9**, e01406-19 (2020).
2. Kiesewalter, H. T. *et al.* Genomic and chemical diversity of *Bacillus subtilis* secondary metabolites against plant pathogenic fungi. *mSystems* **6**, 10–1128 (2021).
3. Lozano-Andrade, C. N., Strube, M. L. & Kovács, Á. T. Complete Genome Sequences of Four Soil-Derived Isolates for Studying Synthetic Bacterial Community Assembly. *Microbiol. Resour. Announc.* **10**, e00848-21 (2021).
4. Yang, N. *et al.* Emergent bacterial community properties induce enhanced drought tolerance in Arabidopsis. *NPJ Biofilms Microbiomes* **7**, 82 (2021).
5. van der Gast, C. J. & Thompson, I. P. Effects of pH amendment on metal working fluid wastewater biological treatment using a defined bacterial consortium. *Biotechnol. Bioeng.* **89**, 357–366 (2005).
6. Piccardi, P., Vessman, B. & Mitri, S. Toxicity drives facilitation between 4 bacterial species. *Proc. Natl. Acad. Sci.* **116**, 15979–15984 (2019).
7. Lozano, G. L. *et al.* Introducing THOR, a Model Microbiome for Genetic Dissection of Community Behavior. *mBio* **10**, e02846-18 (2019).
8. Sun, X. *et al.* Metabolic interactions affect the biomass of synthetic bacterial biofilm communities. *mSystems* **8**, e01045-23 (2023).
9. Niu, B., Paulson, J. N., Zheng, X. & Kolter, R. Simplified and representative bacterial community of maize roots. *Proc. Natl. Acad. Sci.* **114**, E2450–E2459 (2017).
